# Inter-Subject Synchronization of Prefrontal Cortex Hemodynamic Activity During Natural Viewing 

**DOI:** 10.2174/1874440000802010014

**Published:** 2008-04-01

**Authors:** Iiro P Jääskeläinen, Katri Koskentalo, Marja H Balk, Taina Autti, Jaakko Kauramäki, Cajus Pomren, Mikko Sams

**Affiliations:** 1 Department of Biomedical Engineering and Computational Science, Helsinki University of Technology, FIN-02015 TKK, Espoo, Finland; 2 Advanced Magnetic Imaging Center, Helsinki University of Technology, FIN-02015 TKK, Espoo, Finland; 3 Helsinki Medical Imaging Center, Helsinki University Central Hospital, Helsinki, Finland

## Abstract

Hemodynamic activity in occipital, temporal, and parietal cortical areas were recently shown to correlate across subjects during viewing of a 30-minute movie clip. However, most of the frontal cortex lacked between-subject correlations. Here we presented 12 healthy naïve volunteers with the first 72 minutes of a movie (“Crash”, 2005, Lions Gate Films) outside of the fMRI scanner to involve the subjects in the plot of the movie, followed by presentation of the last 36 minutes during fMRI scanning. We observed significant between-subjects correlation of fMRI activity in especially right hemisphere frontal cortical areas, in addition to the correlation of activity in temporal, occipital, and parietal areas. It is possible that this resulted from the subjects following the plot of the movie and being emotionally engaged in the movie during fMRI scanning. We further show that probabilistic independent component analysis (ICA) reveals meaningful activations in individual subjects during natural viewing.

## INTRODUCTION

Human brain mapping studies have traditionally utilized highly simplified and/or artificial stimuli in strictly defined experimental task paradigms to pinpoint the areas processing particular stimulus features and/or participating in specific cognitive operations. While these studies have significantly advanced our understanding of the neural basis of cognition, rapid developments in non-invasive neuroimaging methodology have made it increasingly possible to record and study brain activity also during more natural viewing conditions [1-5].

A recent study showed robust inter-subject correlation of hemodynamic brain activity when subjects freely viewed a 30 minute video clip from the movie “Good, bad, and the ugly” [3]. Specifically, fMRI activations of a given reference subject were used to predict activations in other subjects using a voxel-to-voxel correlation analysis. The sensory and association areas in the temporal, parietal and occipital lobes, as well as the anterior cingulate cortex showed significant intersubject correlation, and peak activations occurred in meaningful parts of the movie, for instance, the fusiform gyrus was activated when faces were shown in the movie, and post-central gyrus activations were seen when hands manipulating objects were shown in the movie [3].

In contrast to the other areas, there was an apparent lack of inter-subject synchronization of hemodynamic activity in frontal cortical areas [3]. This was interpreted by the authors as implying that the type of abstract goal-directed processing inherent to prefrontal cortical areas relates to subjective states that do not occur in synchrony across different subjects. However, since only an isolated 30-minute clip taken from the movie was shown to the subjects, it is also possible that the subjects were not emotionally engaged and/or following the plot of the movie, but rather viewed the clip as a series of loosely related scenes. Here, to specifically study whether intersubject correlation of also prefrontal cortex activity occurs when subjects are following the plot of the movie and are emotionally engaged in the movie, we showed the first 72 minutes of a movie “Crash” (2005, directed by Paul Haggis) outside of the scanner. The last 36 minutes of the movie were then shown in the fMRI scanner. We hypothesized that we would replicate the previous findings of intersubject synchronization of sensory and non-frontal association cortex activations, and that we would additionally see significant intersubject synchronization of the prefrontal areas.

One of the limitations of the intersubject correlation approach is that activations with inter-individual variability cannot be detected using it. In fact, this limitation was speculated to have caused the lack of intersubject synchronization of prefrontal hemodynamic activity when subjects freely viewed the movie clip in the previous study [3]. Therefore, our second objective was to test whether recently developed probabilistic independent component analysis (ICA) can enable meaningful analysis of hemodynamic responses during natural viewing conditions at the individual level. This has been previously suggested to be the case using a more traditional ICA approach [1, 2, 4]. The probabilistic ICA offers easier interpretation of the results obtained, since the analysis determines which independent components (ICs) are statistically significant [6], unlike the more traditional ICA approach where the researcher needs to select the most feasible ICs from amongst all the possible ICs [7].

## MATERIALS AND METHODS

### Subjects

15 healthy right-handed subjects (age range 19–44, seven females) participated in the study, three of whom were discarded from the analysis due to technical reasons/poor data quality. 11 of the remaining subjects were native Finnish speakers, and one was a native English speaker. Only subjects fluent in English were accepted to the study since the language of the movie that served as the experimental stimulus (“Crash”, 2005, Lions Gate Films) was in English. Further, only subjects who had not seen the movie previously were accepted to the study. Ethical approval was granted by the coordinating ethical committee of Hospital district of Helsinki and Uusimaa, the study was run in accordance with the guidelines of the Helsinki declaration, and a written voluntary consent was obtained prior to participation in the study from each subject.

### Experimental Design

Each subjects watched the first 72 minutes of the movie on a LCD screen in a dimly lit room with 5-speaker surround sound settings. The subjects were instructed view the movie freely as if they were in a movie theater. The movie was then discontinued and the subject was prepared for the fMRI scans, which took about 30 minutes. During fMRI scanning the last 36 minutes of the movie were shown in two parts lasting 14 and 22 minutes, respectively, with a waiting time of about 30 seconds in between (due to a technical limitation in the number of slices that can be obtained in a single run in our 3T scanner, and the pause placed to a naturally occurring point in the events of the movie). The movie was projected onto a translucent screen, which the subjects viewed through an angled mirror, and the audio track of the movie was played to the subjects *via* a pneumatic audio system with earplugs. Presentation software (Neurobehavioral systems Inc., Albany, CA) was used to show the movie. The audio track of the movie was delivered/played to the subjects with an UNIDES ADU2a audio system (Unides Design, Helsinki, Finland) *via *plastic tubes through a porous EAR-tip (Etymotic Research, ER3, IL,USA) earplugs.

### fMRI Scanning

A General Electric Signa 3 Tesla MRI scanner with Excite upgrade located in the Advanced Magnetic Imaging Center at the Helsinki University of Technology was used in the present study. An 8-channel head array coil was used. After an initial localizer scan, 45 axial T1-weighted anatomical MRI slices (slice thickness 5 mm, in-plane 2 x 2 mm) were acquired. The subsequent echo planar imaging (EPI) slices were then obtained from identical positions. A total of 630 whole-brain T2* weighted gradient echo EPI volumes (TR = 3400 ms, TE = 32 ms, flip angle 90 degrees, 52 axial slices, 3 mm slice thickness without gaps, 3 × 3 mm in-plane resolution) were acquired during the presentation of the movie in two runs of 14 and 22 minutes (the first two volumes of each run were discarded from the analysis). The subjects were instructed not to move during the scanning session in order to avoid motion artifacts. T2-weighted fluid-attenuated inversion recovery and high resolution 3D T1-weighted structural images were obtained after the subjects had viewed the two movie clips. The continuous scanner noise of the whole-brain EPI was attenuated by viscoelastic mattress inside and around the headcoil and under the subject. The coolant pump of the scanner was switched off during the fMRI scanning in order to further reduce acoustic noise.

### Data Preprocessing

The functional DICOM images were first converted to 4D Analyze format volumes with MRICro (MRICro, 2005). The FSL software (FMRIB Centre, Oxford, UK; http://www.fmrib.ox.ac.uk/fsl/) was then used for further processing. Non-brain structures were first removed from the functional images using BET. The whole-brain images were then realigned with MCFLIRT motion correction. The slices were temporally realigned using interleaved slice timing correction to compensate for acquisition time lags. High-pass temporal filtering option of the FSL package was applied (i.e., linear detrending) and the data were spatially smoothed with a Gaussian kernel of 8 mm full width at half maximum (FWHM).

### Between-Subjects Voxel-to-Voxel Correlation Analysis

In order to be able to compare the voxels of different subjects’ to each other, the preprocessed images were co-registered to the Montreal Neurological Institute (MNI) template. This was done by first registering the functional images to high resolution anatomical images (T1s) and then registering the resulting images to MNI reference template images. Once the images from both of the subjects’ functional runs had been normalized into the common MNI space, the time courses of a given subject’s voxels were used to predict activation in the corresponding voxels of the other subjects. These analyses were conducted using Matlab 6.5 software (Mathworks, Natick, MA). The images in Analyze format were read into Matlab as 4D matrices. Since the data was acquired in two separate scans, the images from the two EPI sequences were combined into a single 64x64x52x626 matrix. After concatenating the data sets, correlation coefficients were calculated between the time series of the reference subject’s voxels and the corresponding voxels of all the other subjects individually. The correlation coefficients were thresholded at *p* < 0.01.

### The Probabilistic ICA

The pICA [6] were conducted on the preprocessed data sets of individual subjects using the Multivariate Exploratory Linear Optimized Decomposition into Independent Components (MELODIC) package of the FSL. The automatic dimensionality estimation option was used in order to reduce the number of and find the relevant independent components from the data sets. Once the ICs were calculated individually for each subject, we attempted to identify similar components between subjects. This was done first by correlating the time series of the ICs of 11 subjects to the ICs of a selected reference subject, and repeated this for every subject. IC pairs showing significant correlations (r>0.3) were inspected visually. The ICs that were observed to be correlated were studied further. We examined what kind of events in the movie elicited highest activations in the voxels belonging to a certain IC by taking the across-subjects average of the IC time-course and traced each time point showing significant activation (p<0.001) back to the stimulus. This was done entirely by visual inspection, as we tried to look for a common factor in the movie that was present at all the time points of highest average activations in a given component’s voxels, taking into account the presumed hemodynamic lag of ~3-5 seconds.

### Self-Evaluation of Emotional Valence and Arousal

After fMRI scanning the subjects were asked to recall their emotional experience while watching the last 36 minutes of the movie in the fMRI scanner. The subjects were taken back to the dimly lit room (wherein they had seen the first 72 minutes of the movie prior to fMRI scanning) and the last 36 minutes of the movie was re-shown to them. Once every 30 seconds the subjects were prompted to evaluate the valence and the arousal of the movie on an ordinal scale from 1 to 5. The subjects were instructed to rate negative valence and low arousal as 1 and correspondingly positive valence and high arousal as 5. The movie kept playing straight through and the subjects were instructed to give their answers to both questions without thinking too long and according to the emotions they recollected having experienced in the scanner. Correlation coefficients were calculated between a given subject’s valence or arousal ratings and the time courses of the subject’s voxels and averaged IC timecourses. The fMRI data were correlated with the emotional self ratings assuming a hemodynamic lag of a single TR.

## RESULTS 

### Between-Subjects Voxel-to-Voxel Correlation Analysis

**Fig. (1):**
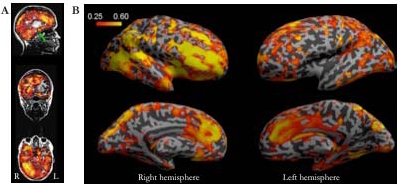
Inter-subject correlations of brain activity during free viewing of the movie clip. A: The across-subjects averaged inter-subject correlation coefficients plotted on sagittal, coronal and axial T1-weighted anatomical scans of a single subject showing the widespread correlations in both cortical and subcortical structures. The green arrow indicates the position of the amygdala. B: The inter-subject voxelwise correlations visualized on the inflated left and right hemisphere cortical surfaces. Note the robust activations in prefrontal and orbitofrontal areas, as well as in the cingulate gyrus during viewing of the movie, and the stronger intersubject correlations in the right than left hemisphere frontal areas.

Significant inter-subject correlations was observed during watching the movie in many cortical and subcortical areas (including the amygdalas). Many prefrontal cortical areas were also significantly correlated across subjects, with right hemisphere predominance (see Fig.**1**). Only relatively small differences in results were observed with different reference subjects, and when correlations were calculated for individual subject pairs.

### The Probabilistic ICA

The number of independent components (ICs) found for each subject using the probabilistic ICA varied between 270 and 340. We were able to identify eight ICs that showed both high correlations and passed the visual inspection of similarity in at least ten of the twelve subjects. ICA segregated many components that were clearly artefacts, but in general these were not the most robustly correlated ones between the subjects. Four ICs that consistently occurred in the subjects were characterized as visual. These components included the primary visual areas V1, V2 (one component per hemisphere) and V5 all mapped into separate components. Single-subject examples of these ICs, as well as an example of a “network” IC consisting of a network of activated frontal and occipital areas, are shown in Fig. (**2**).

### Backtracking the Time Points of Significant Activation to the Events of the Movie

It was not obvious at all which aspects of movie viewing caused the activations in case of the visual and association cortex components. We were also not able to find clear events in the movie that would had explained the inter-subject synchronization in the prefrontal areas. However, two ICs involving activation of superior aspects of the temporal lobe (superior temporal plane, superior temporal gyrus, superior temporal sulcus and middle temporal gyrus) here called “auditory ICs” consistently responded best to movie scenes containing speech. During nearly all parts of the movie where speech was present the time courses of these two ICs showed significantly increased activity compared to baseline. Notably, these ICs showed no increased activity to other types of auditory stimuli such as music or natural non-speech sounds. The increased activity was observed to start approximately 3–6 seconds (2 TRs) after the onset of speech in the movie, which is consistent with the known temporal lag of the hemodynamic response. Fig.(**3**) illustrates the significantly activated time points of these two auditory ICs (ICs with arbitrary numbers of #139 and 264 in this particular subject), with exemplary frames of the movie at the corresponding time points. Even though both of the time courses of the ICs correlated very well with speech presented in the movie, IC #264 showed slightly more speech specificity than IC #139.

### Self-Evaluation of Emotional Valence and Arousal

The subjective ratings of arousal and valence during the 36 minutes of movie viewing (recollected immediately after the fMRI scanning session) significantly varied from the values expected based on the null hypothesis of there being a constant neutral valence (i.e., rating of “3”), and the subjects being non-aroused (i.e., rating of “1”). Further, the changes in arousal/valence coincided with emotion-evoking parts in the plot of the movie (see Fig.**4**). The arousal ratings showed more overall correlation with voxel time series than the valence ratings that failed to show any consistent correlation patterns. Some of the subjects spontaneously reported difficulties with rating the valence, as they felt at times having experienced simultaneously both strong negative and positive valence, thus giving an “average” rating of 3, and at other times lack of specific valence, also resulting in a rating of 3. The arousal ratings correlated with cerebellar areas in practically all subjects and in addition in some cases with prefrontal areas as well. We failed to find correlations between the emotional ratings and the independent components found with ICA.

## DISCUSSION

Cognitive neuroimaging studies have typically utilized specific data analysis models based on a highly specific stimulus sequence and task paradigms used. For instance, periods during which specific auditory or visual stimuli are presented have been contrasted to baseline conditions that do not contain any stimuli. In natural viewing studies, it is not possible to utilize these types of predefined models. Here, we conducted a voxel-wise inter-subject correlation analysis in the vein of [3], which effectively reveals hemodynamic activity that is synchronized during viewing of a movie clip. We also studied whether probabilistic ICA, an improved version of ICA that allows automatic selection of the significant ICs from all the possible ICs for closer scrutiny [6], can be used to study brain activity during natural viewing conditions at the individual subject level.

**Fig. (2):**
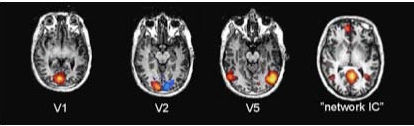
ICs of V1, V2, and V5, as well as an example of an IC containing a network of activated areas encompassing frontal and occipital areas, in a representative subject.

**Fig. (3):**
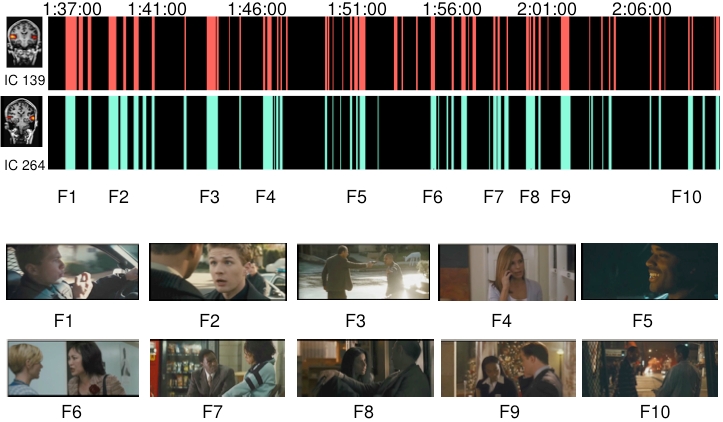
The selective responses of two auditory independent components. **Above:** The time points that differed significantly (p<0.001) from the baseline of the IC’s time series are presented in red and green color. **Below:** Examples of movie scenes (F1-F10) that induced the highest activations. As can be seen, the activation peaks of the ICs seemed to occur when there was speech in the movie. (Movie stills courtesy of Lions Gate Films, 2005.)

Supporting the previous observations [3], we observed highly synchronized responses to the movie across different subjects in the sensory and association cortical areas of temporal, parietal and occipital lobes as well as the anterior cingulate cortex (see Fig.**1**). Even when different reference subjects were used in the correlation analysis the results obtained remained highly similar. Further, the overall correlation coefficients were relatively high. Even though it must be kept it mind that fMRI data always contains some degree of autocorrelation, we were able to observe the correlations with relatively little spatial smoothing (8 mm FWHM in the present study *vs*. the previously used 12 mm [3]). Using the probabilistic ICA, it was possible to reveal activated areas and networks of areas (which may be even more intriguing [8]) in the individual subjects during the natural viewing conditions (see Figs.**2**and**3**), a finding which is also in line with and extends the observations made in previous studies [1, 2, 4, 5].

In contrast to the previous studies, our results revealed significant intersubject correlations in the frontal regions (see Fig.**1**). It was previously reported that the frontal lobe showed very little and usually not at all intersubject correlation whereas more primary sensory processing areas showed significant correlations [3]. It was surmised that this reflected a division of the cortex into areas that respond in a uniform manner to even complex external stimuli and areas whose performance is unique to each individual [3].

There are several possible explanations as to why the frontal areas were synchronized in our study. It is possible that the movie we used was directed in such a manner that individual differences in the subjective experience were not as great as during the video clip that was shown in the previous study [3]. It is also possible that this resulted from the subjects being emotionally engaged in the movie and/or cognitively following the plot of the movie, since we showed the first 72 minutes of the movie outside of the scanner in order to involve the subjects in the movie before commencing with the fMRI scanning.

In the present study, intersubject correlation was more prominent in the right than left hemisphere. Specifically, the hemispheres showed similar correlations in the occipital and temporal lobes, but the frontal regions were much more correlated on the right. In a previous fMRI study, it was observed that right middle temporal cortex, the right posterior superior temporal cortex, and the right inferior postcentral gyrus exhibited stronger activity than the respective left-hemisphere areas during perception of series of still pictures from a movie when they were presented in a coherent order [9].

**Fig. (4):**
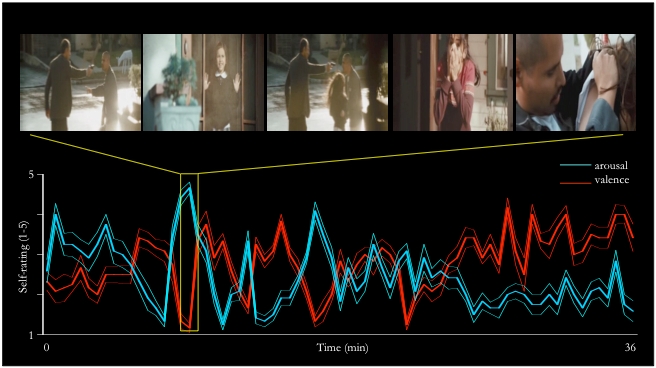
Valence and arousal ratings dynamically changed through out the movie. Here, the arousal (blue) and valence (red) mean (thicker lines) ± SEM values (thinner lines around the thicker lines) are plotted on a timeline, and the part of the movie eliciting highest arousal is depicted above. This particular part of the movie contains a scene in which a man seeking revenge based on a misunderstanding is pointing a gun at the father of a small girl, the girl then runs out of their house to protect her father, her mother is scared about the situation, and the man shoots accidentally at the girl. It then turns out that the gun is loaded with blanks, and the situation is resolved as the father of the girl wonders why there is no wound. Importantly, even though not explicitly tested here, it is possible that if this one minute clip was played in isolation, it would not elicit as strong arousal / valence changes, since the experimental subjects would not be engaged to follow the plot of the movie. Note that the arousal is significantly elevated already in the beginning of the fMRI scanning session. (Movie stills courtesy of Lions Gate Films, 2005.)

It is also possible that the right-hemisphere dominance is explained by the right-hemisphere dominance in emotional processing of visual stimuli [10, 11], since the subjects self-reported being emotionally engaged while watching the last 36 minutes of the movie during fMRI scanning (see Fig.**4**). We could not, however, verify this since the arousal ratings correlated most consistently with cerebellar activity (for corroborating findings, see [12]), and with prefrontal activations only in some subjects, and we failed to see any consistent correlations between the valence ratings and fMRI activity. For the valence ratings, some subjects complained about difficulties in self-reporting valence on a single bipolar scale, since they subjectively experienced negative and positive valence simultaneously. It is a widely held position that positive and negativity affect can be represented on a single bi-polar valence scale [13], and the present findings tentatively support a competing view according to which negative and positive affect are generated independently [14, 15]. The limited number of evaluation time points is an additional potential problem with the emotional ratings in the present study, since the subjects rated their emotions once every 30 seconds adding up to 72 rating points for both valence and arousal. Hence we had no continuous model of the self rated emotional experience. In hindsight, a higher sampling rate, and separate assessment of positive and negative valence, could have made assessment of the emotional state more reliable. In future studies, it would be also useful to monitor autonomic responses elicited by emotions, such as heart rate or galvanic skin response, as additional measures for evaluating the subject’s emotional state.

## CONCLUSIONS

In conclusion, natural viewing study paradigms seem to offer a promising tool to study the intersubject synchronous functioning of even the hierarchically higher-order prefrontal areas. On the other hand, we failed to see any straightforward relationship between the events in the movie and synchronized prefrontal activity. While the movie clip stimulus used in our experiment is much closer to natural conditions than the traditional stimuli used in brain research, it is still quite limited as a research method in that the subjects view the scene but do not actively participate in it. One possible solution to get the subject involved could be using a video game as a stimulus, which would allow study of, for instance, decision making processes in simulated real life contexts.
